# Evaluation of the impact of the COVID-19 lockdown on BMI in children and adolescents with or without obesity

**DOI:** 10.1186/s12887-022-03565-y

**Published:** 2022-08-25

**Authors:** Albane B. R. Maggio, Claudine Gal-Dudding, Xavier Martin, Catherine Chamay-Weber

**Affiliations:** 1 Health and movement consultation, Service of Pediatric Specialties, Division of Pediatric Specialties, Department of pediatrics, gynecology and obstetrics, University Hospitals of Geneva and University of Geneva, 6, rue Willy-Donzé, 1211, 14 Geneva, Switzerland; 2 Adolescent Health Unit, Division of General Pediatrics, Department of pediatrics, gynecology and obstetrics, University Hospitals of Geneva and University of Geneva, Geneva, Switzerland

**Keywords:** COVID-19, Lockdown, Confinement, Childhood obesity

## Abstract

**Background:**

In Switzerland, from March 15th to May 11th 2020, schools and most shops were closed nationwide due to the COVID-19-related lockdown. This cessation of activities may have impacted weight gain in children and adolescents. The aims of our study were to evaluate the effects of the COVID-19 lockdown on the BMI of children and adolescents in treatment for obesity, and to compare its evolution to that of the previous year at the same time, as well as to that of normal-weight children.

**Methods:**

This retrospective study gathered demographic and anthropometric data from subjects aged 6–18 years both with normal weight and with obesity, who attended our hospital clinics at four time points: before and after the lockdown period in 2020, and at the same times of the year in 2019. We used paired t-tests to assess weight, BMI and BMI z-score changes, linear and standard multiple regressions, independent Student’s t-tests or Chi-square tests to compare groups, and Pearson correlation coefficient when appropriate.

**Results:**

Forty-seven children with obesity and 18 normal-weight subjects had complete data for the 4 visits. The mean BMI increased in both groups during the lockdown (obese: + 0.96 ± 1.5 vs. control: + 0.51 ± 0.1), however the increase was significantly more important in the subjects with obesity compared to the same period in 2019 (2019: + 0.33 ± 1.0; mean difference between 2019 and 2020: + 0.63 ± 2.0 *p* = 0.034).

**Conclusion:**

The COVID-19 lockdown had a negative impact on the BMI of youth with obesity. Interestingly we observed extreme changes in this population, which was not the case in normal-weight children. Therefore, families with a child with obesity must be actively supported during these stressful and obesogenic periods of confinement.

## Background

The prevalence of childhood obesity reaches 20–25% in European countries and its treatment has become a great challenge worldwide [1]. All recent studies highlight the multidisciplinary approach necessary to tackle this complex and multifactorial health condition [2, 3]. In 2016, we demonstrated from our cohort that with a specialized multidisciplinary team involving trained doctors, nurses, dieticians, psychologists and physical trainers, we could stabilize and even decrease body mass index (BMI) z-score in 80% cases of children and adolescents with overweight or obesity [4]. However, in our daily practice, we are confronted to ups and downs due to life events that influence weight gain. For instance, we have long since learned that school-related stress (e.g. due to bullying) [5] or summer vacations [6] are punctual risk factors for weight gain in children, as reflected in the literature.

Due to the COVID-19 epidemic that struck the world in 2020, a lockdown has been imposed in Switzerland between March 15th and May 11th 2020. As other obesity specialists [7–9], we hypothesized that the closing of schools and sport clubs, as well as the impossibility of having a regular health monitoring at our clinic, would have a negative influence on the weight of our population of children and adolescents with obesity. Studies in children and adults with obesity have reported changes in lifestyle behaviors during the lockdown, but none monitored their weight [10–14]. Studies that reported weight gain were based on self-reported weight measurements and no standardized/official measures were used [13, 14].

The aims of our study were to evaluate the effects of the COVID-19 lockdown in spring 2020 on the BMI of children treated for obesity in our clinic, and to compare it to the same period in 2019. Finally, we compared their evolution with that of normal-weight children who were followed for other medical reasons during the same periods.

## Materials and methods

### Study design and subjects

This is a retrospective study, embedded in an ongoing cohort study held at the University Children’s Hospital of Geneva.

We analyzed data from two groups of children:

1. Subjects with overweight or obesity (referred to as “subjects with obesity”), aged 6 to 18 years, who attended the obesity clinics in Geneva at two time points in 2019 and in 2020 (4 visits in total): one visit between January 1st and March 15th (i.e. before the confinement) and another one between June 1st and August 31st (i.e. after confinement.
2. Subjects with normal weight (referred to as “control subjects”), of the same age range, who attended other consultations at the same time points in 2019 and in 2020 (4 visits in total). We analyzed anthropometrics of 18 normal-weight children out of the 69 children who had four visits, after exclusion of 14 subjects without signed consent, 13 toddlers who were younger than our population (< 6 years old), 15 underweight (BMI z-score < −1SD) and 9 overweight or obese children (BMI z-score > +1SD).

In normal circumstances, subjects undergoing treatment for their obesity at our clinic are followed up every one to 3 months by a doctor or a nurse and, depending on their needs, by a dietician or a psychologist. A detailed description of our specialized follow-up can be found in a previous publication [4]. During the COVID lockdown, our consultations were closed and our patients were unable to attend their usual appointments. Control subjects were followed at other clinics, such as: gastroenterology clinic for inflammatory bowel diseases, constipation, gastroesophageal reflux, undetermined chronic stomach ache, …; neurology clinic for epilepsy, febrile convulsion, neurodevelopmental diseases, TDAH, …; pneumology clinic for asthma, fibrosis cystic disease, chronic cough, thorax malformations, …; or immunology clinics. We chose not to include the most severely affected patients whose disease could have had a negative influence on their weight evolution, and thus excluded those who were underweight.

### Measures

#### Demographics and anthropometrics

We collected children’s age and gender, as well as body weight (kg) in light clothes (panties and tee-shirt) and height (cm) without shoes. Body mass index (BMI) was calculated as weight/height squared (kg⋅m^− 2^) and z-scores were derived using the World Health Organization references [15]. Underweight was defined as a BMI z-score < − 1SD, normal weight as − 1 to +1SD, overweight as > + 1SD, obesity as > + 2SD and severe obesity as > + 3SD.

#### Statistical analyses

##### Power calculation

To calculate the power of our analyses, we used the BMI change and not the BMI z-score, because we observed that the BMI z-score changes were not related to the amplitude of BMI changes when comparing normal-weight and obese subjects. In fact, especially in 2020, the BMI z-score change was lower in obese children compared to control subjects, despite considerable increase in their BMI. This discrepancy has already been pointed out in other studies that concluded that BMI z-score is inaccurate to track changes in severely obese children and that BMI must be used instead [16–20]. That is why we focused our results and discussion on the BMI changes in our study, even if BMI z-scores are presented.

The number of children in each group (subjects with obesity *n* = 47, control subjects *n* = 18) allows the calculation of the effect size of our study. In the group with obesity, with a difference in BMI change of 0.63 ± 2.0 between 2019 and 2020, the effect size was medium (0.558). In the control group, with a difference of − 0.33 ± 0.1, the effect size was very high (1.0). To detect a difference of BMI change of 0.42 ± 0.37 between obese and control subjects in 2020, the effect size of our study was large (0.997).

##### Statistical analyses

Statistical analyses were performed using the SPSS software 25.0 (Chicago, IL). A descriptive analysis was performed using frequency distributions for the qualitative variables and mean, standard deviation (SD) and range for the quantitative ones (anthropometrics measures). In each group, we looked at the evolution of anthropometric data within each year and between 2019 and 2020. Then we compared the evolutions between the two groups. We used paired t-tests to assess weight, BMI and BMI z-score changes, linear and standard multiple regressions, independent Student’s t-tests or Chi-square tests to compare groups, and Pearson correlation coefficient when appropriate. The differences were considered as significant if *p* < 0.05.

## Results

### Subjects with obesity

#### Characteristics

Characteristics of this group of patients are displayed in Table 1. In 2019, 17% were overweight (BMI z-score 1–2; *n* = 9/47), 59.6% were obese (BMI z-score 2–3; *n* = 28) and 23.4% were severely obese (BMI z-score > 3; *n* = 11). Seventy percent (*n* = 33/47) were under the age of 12 in 2019.

**Table 1:** Characteristics of subjects with obesity and normal weight in 2019 and 2020

	**Subjects with obesity**		**Control subjects**	
Number of subjects:	47		18	
Gender female: n (%)	24 (51.1)		6 (33.3)	
*January to March*				
	2019	2020	2019	2020
Age: years	11.2 ± 2.1	12.2 ± 2.1	10.8 ± 3.3	11.8 ± 3.3
Age range: years	6.3–15.5	7.2–16.5	6.0–15.7	7.0–16.7
Weight: kg	63.6 ± 15.8	71.0 ± 15.4	36.8 ± 12.6	40.2 ± 12.8
Height: cm	151.8 ± 11.6	157.4 ± 11.1	143.9 ± 18.8	148.1 ± 17.4
BMI: kg.cm^− 2^	27.1 ± 3.7	28.3 ± 3.5 ^δδδ^	17.2 ± 1.8	17.8 ± 2.1^δδ^
BMI z-score	2.72 ± 0.8	2.67 ± 0.8	− 0.11 ± 0.5	− 0.06 ± 0.5
*June to August*				
Time in-between both visits: months	4.5 ± 1.5	4.3 ± 1.3	3.4 ± 1.1 ^††^	3.8 ± 0.9
Weight: kg	66.5 ± 15.9	75.2 ± 16.0	38.5 ± 13.6	42.4 ± 13.2
Height: cm	154.4 ± 11.4	159.5 ± 11.1	144.8 ± 19.0	149.7 ± 17.1
BMI: kg.cm^− 2^	27.5 ± 3.7	29.2 ± 4.0 ^δδδ^	17.7 ± 2.1	18.3 ± 2.1 ^δ^
BMI z-score	2.6 ± 0.7	2.72 ± 0.8	0.56 ± 0.5	0.11 ± 0.5
Weight gain between both visits	2.9 ± 2.5 ^***^	4.2 ± 4.7 ^***^	1.7 ± 0.4 ^***, †^	1.6 ± 0.4 ^***, ¥¥^
Weight gain: range	−2.3 – 8.4	−10.1 – 18.4	0.2–6.5	0.2–7.0
Difference of BMI between both visits	0.33 ± 1.0 ^*^	0.96 ± 1.5 ^***,δ^	0.87 ± 0.2 ^*^	0.51 ± 0.1 ^***^
Difference of BMI between both visits: range	−1.9 – 3.1	−3.8 – 6.1	−0.3 – 2.8	− 0.01 – 2.1

Results are expressed as mean and SD or range

Differences of weight gain and BMI during the same year: * *p* < 0.05; ** *p* < 0.01; *** *p* < 0.001

Differences of BMI, BMI z-scores, weight gain and BMI increase between 2019 and 2020 per group: ^δ^ *p* < 0.05; ^δδ^ *p* < 0.01; ^δδδ^ *p* < 0.001

Differences of time in-between visits, weight gain and BMI increase between obese and controls in 2019: ^†^ *p* < 0.05; ^††^ *p* < 0.01; ^†††^ *p* < 0.001

Differences of time in-between visits, weight gain and BMI increase between obese and controls in 2020: ^¥^ *p* < 0.05; ^¥¥^ *p* < 0.01; ^¥¥¥^ *p* < 0.001

*Abbreviation*: *BMI* Body mass index

#### Evolution between pre and post-lockdown periods

In 2019, children and adolescents gained almost 3 kg in the 4-month period, while in 2020 weight gain exceeded 4 kg during the 4-month lockdown. However, the difference was not significant when comparing 2019 and 2020 (*p* = 0.126). We examined the characteristics of the 5 children who lost or gained the most weight. The 5 patients who lost the most weight were older than the rest of the subjects (lost weight: 14 ± 1.7 years old vs. others: 11.9 ± 2.1; *p* = 0.035). Furthermore, the time between the two visits was longer for the 5 children who gained the most weight compared to the rest of the subjects (6.0 ± 0.7 months vs. others: 4.1 ± 1.3; *p* = 0.002). When dividing their weight gain by follow-up time, we found a gain of 2.1 kg per months against − 0.94 kg per months for the one who lost the most (*p* < 0.001). There was no difference either in gender repartition or baseline BMI.

**Fig. 1:**
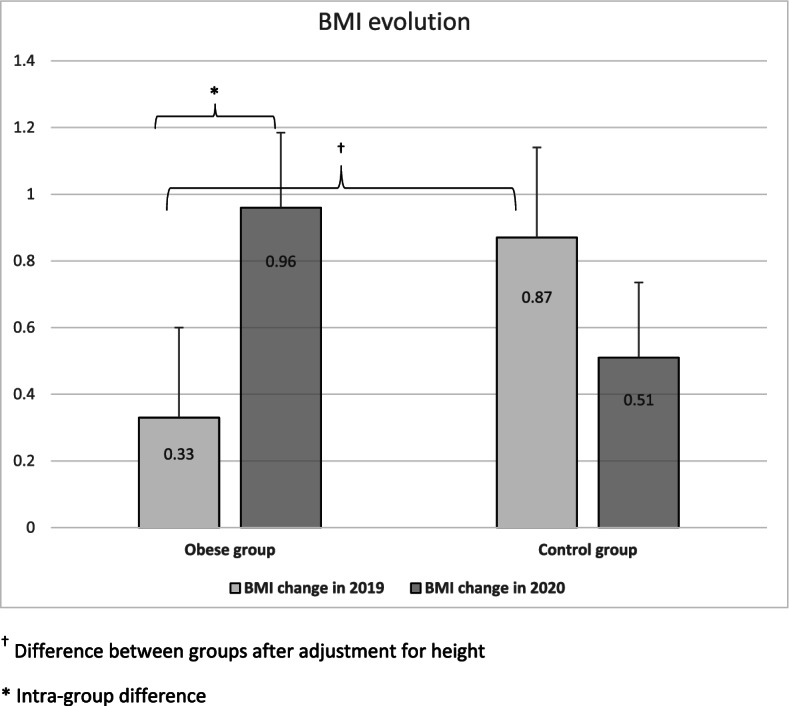
Comparisons of BMI changes between 2019 and 2020. ^†^ Difference between groups after adjustment for height. * Intra-group difference

**Fig. 2:**
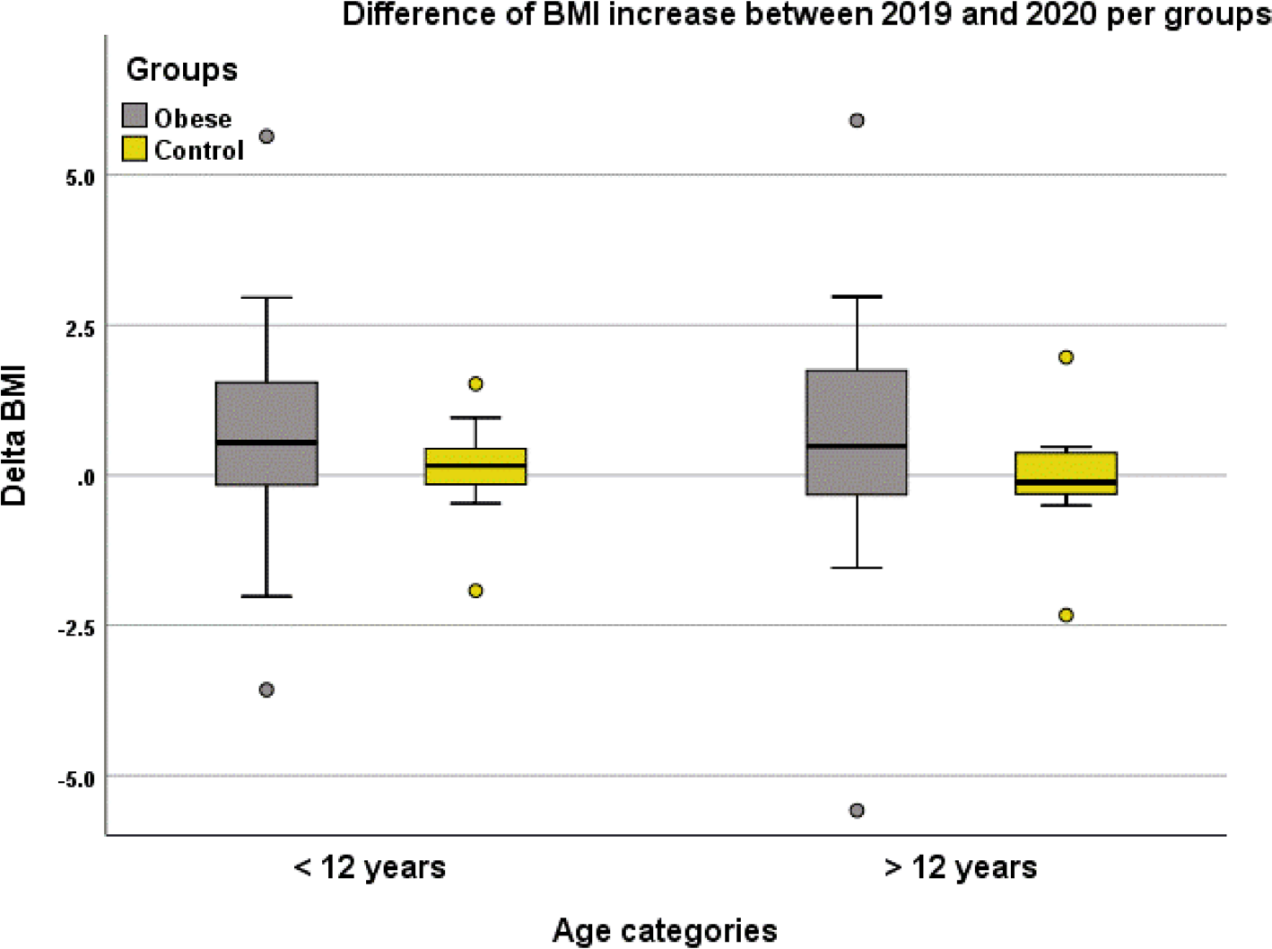
Difference in BMI evolution per groups between 2019 and 2020 according to age categories

As expected, the BMI increased significantly both in 2019 and in 2020 (2019: *p* = 0.023; 2020: p < 0.001). While the increase was of the expected magnitude in 2019 after a 4-month period (BMI + 0.33 kg/m^2^), it was significantly greater in 2020, with a difference between the 2 years of + 0.63 ± 2.0, *p* = 0.034; Fig. 1**.** Figure 2 shows the difference of BMI changes between 2019 and 2020 according to the age category. Although the difference between age categories was not significant, the graph shows a wide range of changes in adolescents with obesity.

When looking at the BMI z-score, it remained stable between the pre and post-lockdown periods both years (2019: − 0.05 ± 0.2, *p* = 0.099; 2020: 0.05 ± 0.2, *p* = 0.145) but was significantly higher in 2020 compared to 2019 (+ 0.1 ± 0.34, *p* = 0.045).

There were no correlations between weight, BMI or BMI z-score evolutions and gender, age or age categories (less than or greater than 12 years) in either 2019 or 2020.

### Control subjects

#### Characteristics

Characteristics of subjects are provided in Table 1. Sixty-one percent (*n* = 11/18) were under the age of 12 in 2019 and there were more boys than girls.

#### Evolution between pre and post-lockdown periods

Normal-weight children gained only 1.7 kg during the studied period in 2019 and 1.6 kg during the lockdown in 2020. The difference was not significant (*p* = 0.368). We also looked at the characteristics of the 5 children who gained or lost the most weight. There was no difference in age, gender or baseline BMI.

As expected, control subjects increased significantly their BMI both in 2019 and in 2020 (2019: *p* = 0.024; 2020: *p* < 0.001; Fig. 1), but even if the magnitude of the increase seemed lower in 2020, the difference was not significant between 2019 and 2020 (*p* = 0.890). Figure 2 shows the difference of BMI change between 2019 and 2020 according to the age category, which was not significant.

Control subjects’ BMI z-scores increased in 2020 (+ 0.17 ± 0.2, *p* = 0.004), while it was stable in 2019 (+ 0.16 ± 0.4, *p* = 0.103).

There was no correlation between weight, BMI or BMI z-score evolutions and gender, age or age categories (</> 12 years) in either 2019 or 2020.

### Comparison between subjects with obesity and control subjects

Mean age, repartition in age categories and gender were comparable between groups (*p* = 0.662, *p* = 0.483, *p* = 0.199; respectively). The time between the two visits was shorter for the control group in 2019 (p = 0.004), but equal in 2020 (*p* = 0.073). Weight gains in 2019 and in 2020 were more important in the subjects with obesity compared to the control group (2019: + 1.3 ± 0.6, *p* = 0.023; 2020: + 2.1 ± 0.8, *p* = 0.01), even after adjustment for the time between both visits. The weight gain per month was similar between both groups in 2019 (*p* = 0.263) and 2020 (*p* = 0.150).

The subjects with obesity increased their height significantly more than the controls in 2019 (*p* = 0.002), but not in 2020 (*p* = 0.309) even after adjustment for age. BMI evolution seemed more important in normal weight in 2019 while we observed the contrary in 2020, but the difference was not significant between the 2 groups either in 2019 or in 2020 (*p* = 0.497; *p* = 0.105). However, after adjustment for the change in height, normal weight children did increased more their BMI in 2019 than children with obesity (*p* = 0.022; Fig. 1). Furthermore, the difference in BMI evolution between 2019 and 2020 was not significant when comparing the 2 groups, even if subjects with obesity seemed to have increased their BMI more in 2020 than in 2019 (subjects with obesity: + 0.63 ± 2.0 vs. control subjects: − 0.33 ± 1.0; *p* = 0.114).

## Discussion

The purpose of this retrospective study was to investigate the effects of the COVID-19 lockdown on BMI on youth with and without obesity.

Indeed, several studies demonstrated that the 2020 lockdown resulted in behavioral and lifestyle changes, especially related to dietary choices and habits as well as in sedentary and physically active occupations [13, 14]. Subjects with obesity seemed particularly at risk since some studies showed that those changes have caused a weight gain of 1.5 to 3 kg in adults suffering from excess weight [10–12], a phenomenon that some authors have called “covibesity” [21]. However, to date, no studies have looked at this issue in children. Therefore the aim of our study was to look at the weight evolution during the lockdown period in children and adolescents with obesity regularly followed in our consultation, and to compare the evolution to the same period the previous year. We were also interested to see if normal-weight children followed for other medical reasons experienced the same difficulties as our population.

Normal growth during childhood involves weight gain and height increase, with an average increase of the BMI of 0.3 points every 3 months. In our study, although we did observe a gain in weight and BMI during the 2 studied periods both in normal-weight youth and in those with obesity, the increase was not of the same magnitude between the two groups.

Indeed, during almost a similar interval, subjects with obesity gained almost twice as much weight as normal-weight subjects, whether it be in 2019 or in 2020. This difference may be explained by several factors. First, this finding may simply confirm the tendency of subjects with obesity to gain more weight than normal-weight children, as suggested by Lagstöm et al. [22]. However, subjects with obesity are known to have more precocious puberty and it is possible that some of them were further along in the puberty process, as might suggest the fact that they grew significantly more (height + 2.6 cm vs. + 0.9 in the control subjects), even after age adjustment. However, since no puberty outcomes were assessed, we can only assume this. Nevertheless, weight gain was more pronounced in the subjects with obesity during the 2020 lockdown period and between 2019 and 2020 with a difference of 1.3 kg, against a difference of only − 0.1 kg in the control group. This difference in weight gain between normal-weight and obese subjects during the lockdown has been already observed in adult studies, with almost identical magnitude of weight gain between the 2 groups as we observed in our study [10, 23]. Similarly, while the BMI increased in both groups in 2019, this increase was more pronounced during the lockdown in 2020 for children with obesity. The difference of BMI between 2019 and 2020 was of 0.6 points in subjects with obesity compared to a decrease of 0.36 points in the control group. It is interesting to note that in 2019, the BMI change was smaller in the obese than in the control subjects when adjusting for the change in height that was different between the 2 groups. This may confirm the positive impact of specific support for children with obesity, as suggested in other publications [24–26]. Furthermore, our results also suggest that it may be more difficult for children and families dealing with weight concerns to maintain their efforts when the environment and living conditions change drastically, such as during the lockdown we experienced in 2020, especially when no medical follow-up can be provided, as was the case in our hospital.

Another interesting result related to the weight range or BMI changes in our study was that subjects with obesity tended to display more extreme changes than controls, especially in 2020. Some patients took advantage of this special circumstance to improve their diet and spend more time exercising, losing as much as 10 kg, while others were unable to stick to our recommendation, gaining up to 18 kg of weight. This may reflect the difficulties encountered by our population suffering from obesity to regulate their weight. The subjects who lost the most weight were older than the others and one explanation could be that adolescents no longer had the possibility to buy fatty and sugary foods after school or during their lunch break. Not surprisingly, the ones who put on the most weight had a longer interval between medical visits. This can have several explanations. First, it is known that motivation lasts only a few weeks and that it may be difficult to maintain an effort when medical appointments are as far apart as 6 months. Secondly, children who knew they had gained weight might have delayed their visit after the end of lockdown in an attempt to stabilize their weight before their next appointment. They might also have been afraid of catching the COVID-19 disease on their way to the hospital using public transports or during their medical visit. Stress being generally recognized as a risk factor related to excessive weight [27], it is possible that anxious patients were negatively impacted by pandemic-related stress, which might have caused, among other consequences, eating disorders to appear or worsen [28–30]. Normal-weight children did not show such extreme changes, as they probably struggled less with food choices or quantities and were more prone to move even when their usual physical activities were cancelled.

This study has some limitations. First, the sample size is small and we could probably have seen a more significant difference between the groups if it had been larger. However, to date, no other study has actually measured and compared weight as precisely at 2 years interval. Secondly, we did not look at the relationship between weight and lifestyle changes in the present study, however, we investigated this outcome in a group of adolescents in different study which results will be published soon (manuscript submitted). Finally, the normal-weight group was composed of children with medical health conditions and not healthy ones, which may have influenced their weight evolution. However, this bias was minimized when we compared 2019 to 2020 in the same children. Furthermore, we excluded underweight children to ensure that seriously ill patients were not included in the sample and we controlled the normal staturo-weight evolution of the selected subjects to make certain that their disease had no significant impact on it. Besides, the vast majority of our subjects were suffering from minor afflictions such as chronic cough, reflux or constipation, conditions on which the lockdown would not have had much influence.

## Conclusion

The COVID-19 lockdown is known to have a negative impact on lifestyle behaviors and our study confirmed an excessive weight gain in children and adolescents with obesity. Furthermore, changes were more polarized in both weight gain and weight loss in this population, which demonstrates the complexity of obesity management. Therefore, families with an overweight or obese child or adolescent must be more actively supported during this stressful and obesogenic times when all their bearings have been disrupted.

## Data Availability

The datasets used and/or analyzed during the current study are available from the corresponding author on request.
